# Reallocation of resources between generations and genders in the market and non-market economy. The case of Italy

**DOI:** 10.1016/j.jeoa.2014.09.003

**Published:** 2015-04

**Authors:** Marina Zannella

**Affiliations:** Institute of Mathematical Methods in Economics, Vienna University of Technology, Austria

**Keywords:** Intergenerational relationships, Gender, Economic transfers, Household production, Time use

## Abstract

In this article the National Transfer Accounts (NTA) method is used to develop a comprehensive account of resource reallocations between population members in Italy, encompassing the age and the gender perspective, the public and the familial institutional sectors as well as the market and non-market dimensions of the economy. The inclusion of the non-market economy, referring to household and care time, allows for an insight into the gender division of labour and the strength of intergenerational obligations in the Italian *familistic* welfare regime. Results highlight the existence of large flows of resources within the family both between genders and toward young generations, with men and women giving rise to considerable monetary and time transfers, respectively.

## Introduction

Increasing life expectancies and low fertility are determinants of population ageing in industrialized countries. This process appears to be particularly severe in Italy, which shows the second highest share of population aged 65+ (21.2%) and old-age dependency ratio (32.7%) among European countries (Eurostat, 2014a). The scenario is expected to exacerbate in the future: according to Eurostat projections for 2050, the old-age dependency ratio will increase by 60% (Eurostat, 2014b).

Ageing poses relevant questions in terms of intergenerational relationships and equity, which are at the heart of the challenges contemporary welfare states are faced with. The debate on intergenerational equity has been mostly focusing on the public sector, with particular regard to fiscal sustainability (e.g. Auerbach et al., 1994). The main argument is whether the growing cost of old-age pensions and health care would excessively bias public transfers and overburden the working-age population, giving rise to a “generational conflict” (Preston, 1984). Nevertheless, a large body of literature has stressed the key role of families in the redistribution of resources across generations, highlighting the existence of strong ties and solidarity (for a review see Arber and Attias-Donfut, 2000).

The National Transfer Accounts (NTA) project (www.ntaccounts.org) has been the first effort to comprehensively measure and analyze intergenerational reallocations, including both the private and the public sector. The NTA method (United Nations, 2013) is based on the disaggregation of macro-economic measures by age and provides information on what people produce, consume, save, transfer and receive at different stages of the life course. Introducing age into national accounts, NTA greatly contributes to shed light on the “generational economy” that is the combination of mechanisms and institutions used to finance consumption at different ages and the contract, either formal or informal, regulating intergenerational economic relationships and reallocations (Lee and Mason, p. 7, 2011)

The “generational contract” (Bengtson and Achenbaum, 1993; Walker, 1996) relies, in turn, on a gender contract, “*a set of implicit and explicit rules governing gender relations which allocate different work and value, responsibilities and obligations to men and women and is maintained on three levels – cultural superstructure – the norms and values of society; institutions – family welfare, education and employment systems, etc.; and socialisation processes, notably in the family*” (European Commission, 1998). Gender division of paid and unpaid work is virtually universal, albeit at drastically different levels across countries. Women, beside their increasing participation in the labour market, continue to play a key role in household production generating many resources, in the form of goods and services, which are distributed across the family to meet the needs of its members. Despite its undeniable social value, unpaid domestic work and, thus, non-cash familial contribution to the welfare, is not recognized as an economic activity, and consequently, is invisible in the System of National Accounts ((SNA) United Nations, 1953).

The exclusion of household production from SNA has long been criticized for underestimating the value of total production and, in particular, of that of women who have been traditionally committed in housework and family care. A main concern regards the perpetuation of gender inequalities, which is supported by the very strong argument that “*there is a very simple equation operating here: if you are invisible in a nation’s economy, you are invisible in the distribution of benefits*” (Waring, 1999). The SNA revision (United Nations, 1993) has partly embraced those criticisms, suggesting the development of satellite accounts for household production. The idea has been reinforced by the United Nations *Fourth World Conference on Women*’s final recommendations (United Nations, 1995) toward national and international statistical organizations to develop time use surveys (TUS) as a tool to measure and evaluate unpaid domestic work. In recent years, thanks to the increasing availability of TUS, a number of scholars have made important efforts in order to evaluate the extent of household production and to develop satellite accounts (e.g. Goldschmidt-Clermont, 1999; Ironmonger, 1996; Landefeld and Mc Culla, 2000; Holloway et al., 2002; Abraham and Mackie, 2005; Krueger et al. 2009).

Household satellite accounts provide us with aggregate measures of unpaid domestic work, and thus ignore the age dimension. However, comparative studies on gender differences in time allocation have highlighted that household production varies significantly during the life course both for men and women, especially in correspondence of key events such as having a child or retirement (Apps and Rees, 2005; Anxo et al., 2007; Zagheni and Zannella, 2013). Recently, within the NTA project, a method has been introduced (Donehower and Mejia-Guevara, 2012) with the twofold objective of further disaggregating measures of market-based intergenerational reallocations by gender and accounting for non-market transfers within the family. The result is the creation of two satellite accounts for age and gender referring to the market and non-market economy, respectively, the latter termed *National Time Transfers Accounts* (NTTA).

This article aims to develop a systematic and comprehensive account of resource reallocations between population members for Italy in the year 2008, encompassing the age and the gender perspective, the public and the private sector and the market and non-market dimensions of the economy. For this purpose, the NTA methodology has been used, ensuring the possibility for the future to compare the results with those of other participating countries. In particular, four main objectives have been established: (i) to estimate intergenerational reallocations occurring within the market, namely constructing NTA for Italy; (ii) to disaggregate NTA measures by gender; (iii) to build an account of non-market transfers across generations and genders within the family, namely constructing NTTA for Italy; (iv) to combine the information related to the market and non-market economy as well as the age and the gender dimension to obtain a complete picture of the economy and of its related redistributive system.

The article is organized as follows: Section ‘Principal data and methods’ describes the principal data and methods, distinguishing between those used for market and non-market reallocations. Main results are presented in Sections ‘The Italian age reallocation system: principal results for National Transfer Account’, ‘Introducing gender into National Transfer Accounts’, ‘Accounting for non-market reallocations within the family: National Time Transfer Account’ and ‘Completing the picture of the Italian age and gender reallocation system: some considerations on total economy’. More precisely, Section ‘The Italian age reallocation system: principal results for National Transfer Account’ shows results for the Italian age reallocation system in the market economy, which are extended to gender in Section ‘Introducing gender into National Transfer Accounts’. Section ‘Accounting for non-market reallocations within the family: National Time Transfer Account’ presents estimates of household production, consumption and non-cash familial transfers. Results for the market and non-market economy are then combined in Section ‘Completing the picture of the Italian age and gender reallocation system: some considerations on total economy’, providing a complete picture of the Italian age and gender reallocation system. Section ‘Completing the picture of the Italian age and gender reallocation system: some considerations on total economy’ also presents an application, using demographic projections and estimated age profiles of production and consumption, to analyze the possible economic consequences of future ageing. Finally, Section ‘Discussion and conclusions’ discusses the cross-sectional picture of the economic relationships across generations and genders in Italy for the year 2008, offering an insight on the underlying generational and gender contracts.

## Principal data and methods

The economic life cycle, defined by age patterns of consumption and production, is a key concept in the NTA approach. The difference between consumption (*C*) and labour income (*Y^l^*) at each age (*x*) provide us with a measure of the life cycle deficit (LCD) or surplus (LCS). A positive value of this difference indicates the existence of a deficit that must be funded through intergenerational reallocations. Conversely, a negative value indicates a life cycle surplus that can be either transferred to deficit ages or saved. In other words, the LCD or LCS must equal the age reallocations, composed by transfers and asset-based reallocations:

(1) $$\underset{\mathrm{LCD}.\mathrm{or}.\mathrm{LCS}}{\underset{︸}{C(x)-Y^{l}(x)}}=\underset{\mathrm{Transfers}}{\underset{︸}{\tau^{+}(x)-\tau^{-}(x)}}+\underset{\mathrm{Asset}-\mathrm{basedreallocations}}{\underset{︸}{Y^{A}(x)-S(x)}}$$

where *Y^A^* is asset income, *S* is saving, *τ*^+^ and *τ*^−^ are transfers received and paid at each age *x*.

Age reallocations are mediated by both the public and the private sector. The latter consists of transfers between households (also termed inter-households transfers), direct or mediated by non-profit institutions, and transfers within the household (or intra-household transfers). However, in Italy, virtually all private transfers are represented by those occurring between members of the same household. Therefore I will refer to private transfers as familial transfers in this article.

Estimating NTA age profiles is a fairly complex process which requires several steps. First, per-capita age profiles are estimated relying on micro-level survey or administrative data. In a second step, aggregate age profiles are calculated by multiplying the per-capita values with the corresponding population size. Hence, at the aggregate level, age profiles reflect both population age structure and per-capita age profiles which, in turn, are largely influenced by the interaction of contextual and behavioural factors (Lee and Mason, 2011). Then, if necessary, age profiles are smoothed using Friedman’s Super Smoother (Friedman, 1984).^1^ Finally, age profiles are adjusted using a multiplicative factor to ensure their consistency with SNA measures, e.g. the sum over all age groups must equal the corresponding aggregate in SNA.

Age and gender reallocations occurring within and outside the market boundaries, given their different nature, require specific treatments and procedures. For this reason, the principal data and method used to construct NTA and NTTA are reported separately in the following sub-sections.

### Market-based reallocations, *National Transfer Accounts*

Constructing NTA is highly data demanding and requires the combination of several different data sources. Therefore, the temporal reference for this article (year 2008) was chosen according to the most recent homogeneous data available. Macro-economic aggregates are taken from the following sources: Sector Accounts (Eurostat, 2008a), Final Consumption Expenditure of Households by Consumption Purpose (Eurostat, 2008b), General Government Expenditure by Function (Eurostat, 2008c) and the European System of Integrated Social Protection Statistics ((ESSPROS) Eurostat 2008d). Estimates of private consumption by age are largely based on Istat (2008a) micro-data from the Household Budget Survey (HBS), while estimates for public consumption are mainly based on administrative records. Labour income includes the compensation of employees (labour earnings, benefits, taxes paid to the government on behalf of employees) and the labour’s share of entrepreneurial income. Age profiles of labour income are estimated using micro data sources provided by Istat (2009)^2^ in the European Income and Living Condition Survey (EU-SILC). Public transfer inflows consist of benefits received through the social protection system and public consumption. Age profiles of social benefits are estimated using administrative data, micro data from EU-SILC and data provided by ESSPROS. The estimates of public transfer outflows are based on micro-data from EU-SILC and the Survey for Household Income and Wealth ((SHIW) Banca d’Italia, 2008). Private transfers consist of inter-household transfers, direct or mediated by non-profit institutions, and intra-household transfers. Transfers between households are directly estimated from EU-SILC micro-data. As there is no available information on transfers occurring within the household, they must be estimated indirectly. More precisely, intra-household transfers are estimated as the balancing item at each age between private consumption and disposable income. Asset-based reallocations are defined as asset income minus saving. Public asset income and public savings are distributed according to age profiles of taxpayers (public transfer outflows). The age profiles of private asset-based reallocations are estimated using micro data from SHIW.

Disaggregating NTA by gender requires the same data sources and procedures already described for “traditional” NTA but estimating sex-specific age profiles instead of only age-specific profiles. Since SNA does not provide macro-economic aggregates by gender, sex specific age profiles are adjusted in order to be consistent with SNA as well as “standard” NTA aggregates.

### Non-market reallocations, *National Time Transfer Accounts*

NTTA sex-specific age profiles of production, consumption and transfers build on micro data from the most recent Italian Time Use Survey (Istat, 2008b) with a sample of 44,606 individuals and 18,250 households. TUS includes three data files: the individual file, the daily diary and weekly diary.^3^ The daily diary consists of time data collected through the diary technique: respondents record time use during the previous 24 h in their own words. Time diaries are randomly distributed across the days of the week to all household members aged 3 years and over.^4^ Diaries provide extremely detailed information, including: description of the main activity carried out by the respondent, the possible presence of a parallel secondary activity, the location where the activity was performed and, if applicable, the presence of another person.

The first step to build NTTA consists in identifying, in our survey, unpaid domestic and family care activities with an economic value, namely household production. For this purpose, a set of activities was selected relying on the third-party criterion (Reid, 1934), i.e. whether it is possible to pay somebody else to perform the activity. More specifically, I refer to the following activities: food management, household upkeep, making and care for textiles, gardening and pet care, construction and repairs, shopping and services, household management, child care, assistance given to an adult household member, travel related to household and care (e.g. travel related to shopping and services or child care), informal help to other households (consisting in the same activities listed above but performed outside the household mostly as a help to not co-resident relatives). Then, monetary values are assigned to household productive activities on the basis of the specialist replacement method, i.e. using a specific wage for each activity instead of a general wage for all the activities. Specific wages are found in the National Collective Agreement for Domestic Workers (2007).

Time use for unpaid production activities is visible in TUS and can therefore be directly estimated from the survey micro data, as the mean time dedicated to the selected activities by sex and age. On the other hand, no information exists about how the time produced is consumed. Therefore, time consumption is estimated through assumptions. The overall time produced within the household is assumed to be equal to the overall time consumed, since no time savings are possible. Time consumption is assumed to vary with age but not with gender. Furthermore, productive activities are distinguished in two main kinds: general and age-targeted activities. The former benefit all members of the household (e.g. cleaning, cooking etc.), while the latter benefit specific age groups (i.e. child care and adult care).

Time consumption for household *j* (*C_j_*) is expressed as a linear function of the number of its components (*N_j_*) by age *x* (see Mankiw and Weil, 1989), with age limits (*a* and *b*) varying according to the nature of the considered activity:

(2) $$C_{j}=\overset{b}{\underset{x=a}{\sum }}\alpha (x)\cdot N_{j}(x)$$

where *a* = 0 and *b* = 90 for general activities; *a* = 0 and *b* = 17 for child care; *a* = 18 and *b* = 90 for adult care.^5^

Parameter estimates^6^ (reported in Tables A1–A3 in the Appendix) are used as weights to assign the production of activities within the household to the consumption of its members by age. Hence, sex specific age profiles of consumption by typology of activity are estimated as the corresponding weighted mean values^7^ over all the households. Finally, the resulting profiles are applied also to the activities produced outside the household, i.e. informal help to other household, since the age pattern of consumption for these activities is assumed to be similar to that of the corresponding activities produced within the household.

As in NTA standard accounts, the difference between consumption and production at each age provides a measure of the life cycle deficit or surplus and must equal age reallocations, which in NTTA consist only of familial transfers since the state does not transfer time directly and, as already mentioned, no savings are possible.

## The Italian age reallocation system: principal results for National Transfer Account

**Fig. 1:**
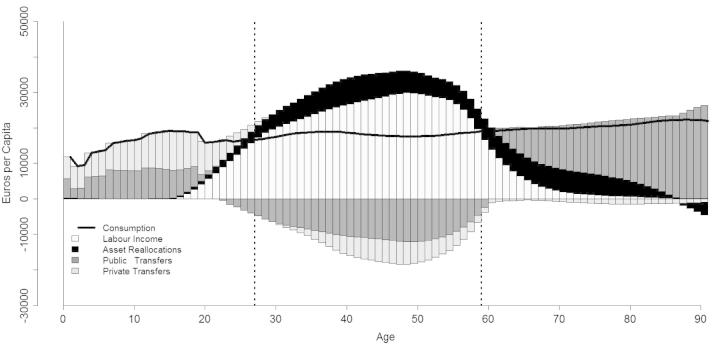
The economic life cycle and age reallocations, both genders, Italy 2008.

*Source:* Own elaborations on Banca d’Italia (2008), Eurostat (2008a,b,c,d), Istat (2008a, 2009) and various other sources.

Fig. 1 presents the economic life cycle (LC) and the intergenerational reallocation system for Italy (year 2008), including age profiles of labour income, consumption, asset reallocations, private and public transfers. Labour income shows a reverse U-shape. It begins at age 15^8^ and grows fast during young and prime adult ages, reaching a peak of about 30,000 euros at 48 years, then it falls rapidly around age 55. Per-capita age profiles of consumption encompass public and private expenditure. Public and private consumption make up 29% and 71% of total consumption, respectively. Public consumption is made up 19% by education, 35% by health and 45% by public goods. On the other hand, education and health constitute only 4% of private consumption, while the remaining 96% are residual expenditures for current consumption and durables. Consumption shows a peak at age 0, mainly motivated by birth-related health costs. During younger ages, it is characterized by a stepped pattern reflecting public education expenditure. It falls rapidly in correspondence of the end of secondary education and then steadily increases with rising age again.

Not surprisingly, the are two broad age groups for which consumption exceeds the labour income, children and elderly persons, and one age group recording a surplus during working ages. However, the specific age limits of these deficit and surplus groups vary significantly across societies, reflecting the underlying economic, institutional and cultural setting. In Italy, the life cycle deficit (LCD) is positive from age 0 to age 26, while that of elderly persons begins at age 59. Also the kind of reallocations financing the deficit groups vary considerably with age, reflecting both the changing nature of needs during the life path and specific features of the welfare system. In virtually all settings the LCD of children is mainly financed by families, albeit to a different extent; while, the support system for elderly people highlights the existence of more relevant differences across countries (see Lee and Mason, 2011). Italian children are supported by 60% through private transfers, 32% through public transfers and 8% through asset-based reallocations. Youth dependency lasts longer within the family than within the state: young people are net beneficiaries of public transfers until approximately age 20, while of familial transfers until age 30. On the other hand, the LCD in old age is mainly financed by public transfers (67%), 28% are asset-based reallocations and only 5% are private transfers.

## Introducing gender into National Transfer Accounts

**Fig. 2:**
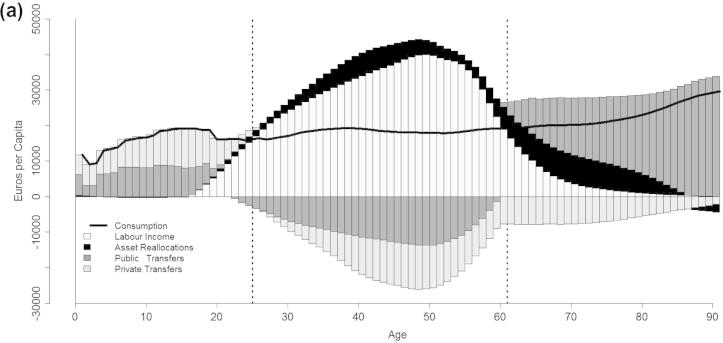

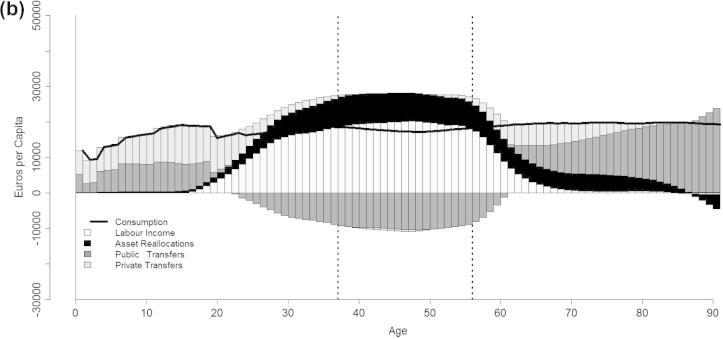
(a) The economic life cycle and age reallocations, Men, Italy 2008. (b) The economic life cycle and age reallocations, Women, Italy 2008.

*Source:* Own elaborations on Banca d’Italia (2008), Eurostat (2008a,b,c,d), Istat (2008a, 2009) and various other sources.

The inclusion of the gender dimension in the analysis of age reallocations within the market is particularly relevant for Italy, which shows one of the lowest female employment rate in Europe (Eurostat, 2014a) with less than half of the active female population participating in the labour market. As expected, the disaggregation of the economic life cycle by gender (Fig. 2a and b) points out the existence of significant differences both in the qualitative shapes and levels of labour income. Labour income starts to rise earlier for men compared to women and increases rapidly with age, peaking at 49 years with a value of 40,000 euros. On the other hand, women compared to men show considerably lower levels as well as a rather flat age pattern of labour income during most of their working life, with a maximum value of 20,000 euros (half of that of men) reached at 47 years. Gender differences persist also during the latest stages of the life cycle: men labour income at old ages lasts longer compared to women as an effect of self-employment.

With respect to the market economy, male young dependency, namely the existence of a life cycle deficit given by a positive difference between consumption and production, lasts until age 24 while old-age dependency starts at age 60 and over (Fig. 2a). Thus, men have a life cycle surplus span of virtually 40 years. On the other hand, women on average are not able to finance their own consumption until age 37 (Fig. 2b). Then, after a surplus lasting for about 20 years, the deficit turns positive again at age 56.

It is noteworthy that Italian women, despite their low employment rates, are able to finance their consumption by the mean of labour income for a period of virtually 20 years. This is mostly explained by the existence of a selection effect on women labour market participation: lower levels of remuneration for women compared to men may encourage couples to opt for a male-breadwinner family model, especially in households with young children. Therefore, less qualified women may decide not to enter the labour market, or to leave it prematurely. This seems to be confirmed by data on the gender pay gap ((GPG) Eurostat, 2014c), 9 showing that the two down-top European countries in terms of female employment rates, Malta and Italy, are also among those with the narrowest earnings differential between genders.

Differences in sex-specific age profiles of labour income are reflected in age reallocations and, particularly, in those occurring in the form of public and private transfers. With regard to net public transfers men and women have similar qualitative age patterns but with relevant quantitative differences, with the exception of young ages during which they receive the same through education and health programs. During working ages, net public transfers are negative for both genders, indicating that they pay more than what they receive through the public sector, although the values are higher for men compared to women. At older ages, net transfers turn positive and grow rapidly, mostly as an effect of retirement pensions but with significant higher amounts received by men.

At young ages men and women show similar patterns also in the case of net private transfers: they are both dependent from older member of the families to finance their consumption. Then, after age 25, their behaviours drastically diverge: net private transfers are negative for women during virtually all their life course, while those of men are positive from age 26 and over. Therefore with regard to market economy, adult men and women are, on average, net contributors and net beneficiaries within the family, respectively.

## Accounting for non-market reallocations within the family: National Time Transfer Account

**Fig. 3:**
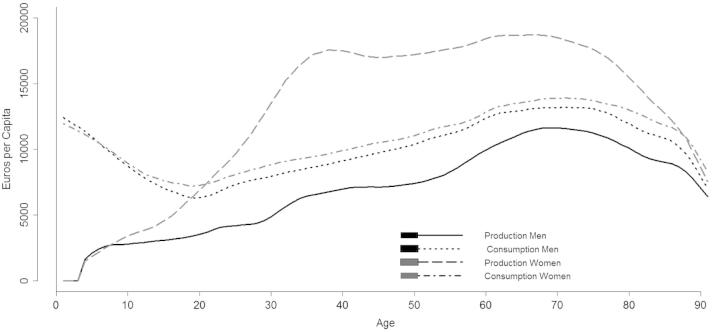
Non market economic life cycle by gender, Italy 2008.

*Source:* Own elaborations on Istat (2008b) and National Collective Agreement on the Regulation of Domestic work (2007).

Considering non-market economy changes the picture of gender and intergenerational relationships drastically (Fig. 3). It should be recalled that time consumption is assumed to be independent of gender and that it is estimated at the household level as a linear function of the number of household components by age (see Eq. (2)). This is clearly reflected in the existence of similar shapes of consumption for men and women, although if with slight different levels which have to be considered as an effect of households composition. Therefore, gender differences in the non-market economic life cycle and, consequently, in net transfers mostly depend on production.

Household production highlights the existence of some common patterns between genders. First, it increases with age, peaking at 66 years for women and 68 years for men, and starting to decrease only after age 80. Second, its age profiles are shaped by two bumps, corresponding to childrearing and retirement ages, respectively. Thus, contrary to what happens in the market, the old population continues to be active in non-market production even during the latest stages of the life cycle. However, the high levels of domestic work produced by elderly persons have to be considered mostly as an effect of the greater amount of time spent at home after withdrawing from the market.

Despite the existence of some similarities, women and men show pronounced differences in non-market production during the entire life course. Already at young ages, female children contribute more to domestic chores compared to their male peers. Results for younger ages suggest the existence of a cultural influence on the division of unpaid domestic work: both male and female children are involved in compulsory education and hence, gender differences at this stage of life cannot be explained as the result of different decisions regarding time allocation to productive activities. Afterwards, female production grows very rapidly until it reaches its first hump, between age 30 and 40, to subsequently stabilize at very high levels (on average more than 17,000 euros per year, corresponding to approximately 8 h of domestic work per day) during virtually all the remaining lifetime. On the other hand, men compared to women increase their production with age less significantly and at a slower pace until retirement.

The presence for both genders of a bump between age 30 and 40 is a common result in comparative studies on gender differences in time allocation, highlighting the great impact of a young child on parental time dedicated to house chores and child care activities (e.g. Apps and Rees, 2005). Nevertheless, the effect is significantly stronger for women than for men. Those studies have shown that as children grow older, especially after reaching school age, women tend to gradually reduce the time devoted to unpaid domestic work and to increase that spent on market work. However, this is not the case of Italy where age profiles of market and non-market work do not present significant changes during the life course: labour income does not experience a significant recovery and household production continues to show very high values. Results, in line with those reported for Italy in Anxo et al. (2007), suggest the existence of a strong gender specialization and the predominance of families oriented toward a male-breadwinner organization.

**Fig. 4:**
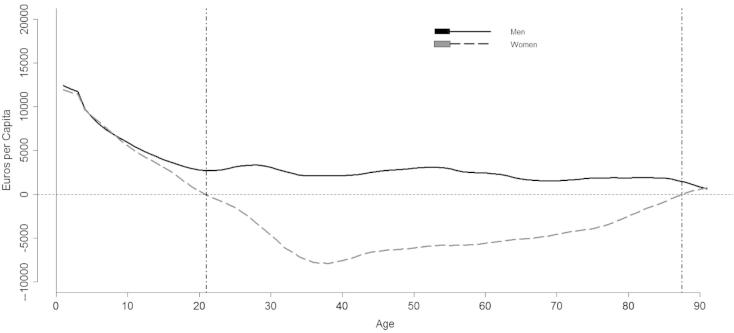
Net familiar non market transfers by gender, Italy 2008.

*Source:* Own elaborations on Istat (2008b) and National Collective Agreement on the Regulation of Domestic work (2007).

Looking at differences between non-market consumption and production at the early stages of life, both genders show a life cycle deficit (Fig. 4). In other words, children consume more domestic time than they produce and therefore depend on time transfers from older members of the family to meet their needs.

After this initial phase, gender differences become evident: women from age 20 and over produce more time related to domestic activities then they consume, while men consume more domestic time then they produce during the entire life course. Consequently, the male non-market deficit is entirely financed by women. Indeed, it possible to assess the existence of a reverse gender pattern between the market and the non-market economy. Nevertheless, women have a market surplus which allows them, at least, to finance their own consumption during a period of their life, which is not true for men in the non-market economy, within which they are completely dependent upon women

## Completing the picture of the Italian age and gender reallocation system: some considerations on total economy

**Fig. 5:**
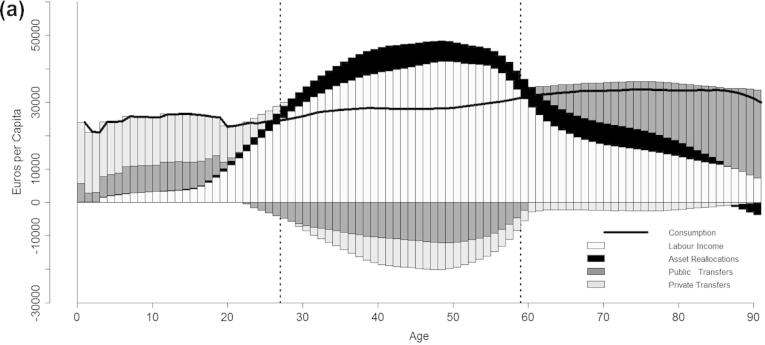

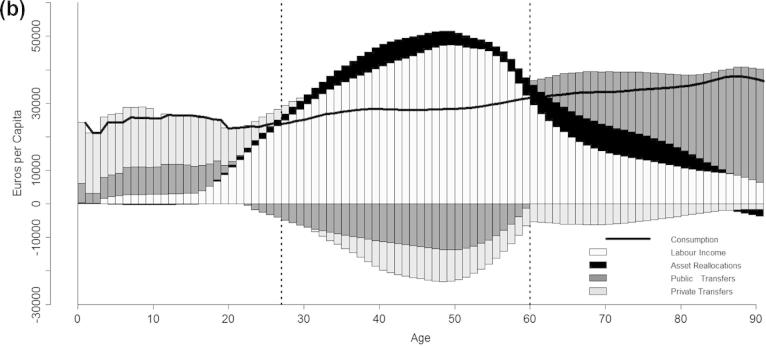

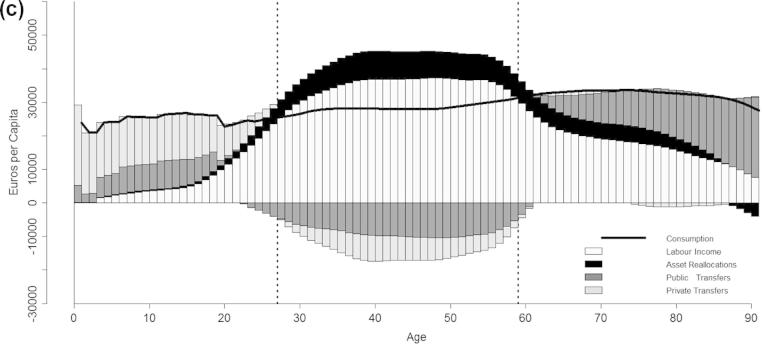
(a) Total economic life cycle and age reallocations, both genders, Italy 2008. (b) Total economic life cycle and age reallocations, Men, Italy 2008. (c) Total economic life cycle and age reallocations, Women, Italy 2008.

*Source:* Own elaborations on Banca d’Italia (2008), Eurostat (2008a,b,c,d), Istat (2008a,b, 2009) and various other sources.

As already mentioned, the strongest effect of the inclusion of the non-market sector in the economy has to be found in production. With reference to the whole population, non-market production raises the value of the total output of economy from approximately 830 to 1450 billion of euros, with an increase of 75%. However, as already highlighted, genders behave very differently in this regard: household production raises the value of production by 36% for men and 144% for women. In order to obtain an in-depth understanding of the generational and gender economy, aggregate values of surplus and deficit ages by gender and economic sector (Table 1) as well as per-capita sex-specific age profiles of the economic life cycle and age reallocations (Fig. 5a–c) are reported.

With regard to total population the levels of surplus/deficit change significantly with the inclusion of unpaid domestic work but the age borders at which the LCD changes from positive to negative remain rather constant. However, men slight reduce while women significantly increase the span of their life cycle surplus (Fig. 5a–c). At the aggregate level (Table 1),^10^ the inclusion of non-market economy raises the value of the youth deficit (compared to that in market economy) by 27%, even though the value for women falls to 22% due to their early contribution within the household. Total surplus of central ages increases by around 20% but, as already highlighted, the rise is totally due to women’s gains in LCS (+95%) while men still decrease their value by 22%. Finally, the economic dependency of elderly persons slightly decreases but, once again, the result is explained by women, while older men show an opposite pattern at older ages, raising the value of their deficit.

Differences in life cycle deficit and surplus are reflected in age reallocations (Fig. 5a–c). It should be recalled that the inclusion of non-market production only affects familial transfers. Transfers to children grow considerably both for male and females. The strongest effect is found at age 0 with an increase in the per-capita value from approximately 7000 to 19000 euros. As an effect of the introduction of non-market economy, familial transfers continue to be positive for men until age 30 while they turn negative at age 27 for women. During central ages, women and men transfer more and less resources, respectively, within the family in total economy compared to market economy. With regard to the total population, transfers from elderly persons toward other ages increase slightly, which is the combined effect of a decrease in the amount of transfers received by women and a decrease of that paid by men.

**Fig. 6:**
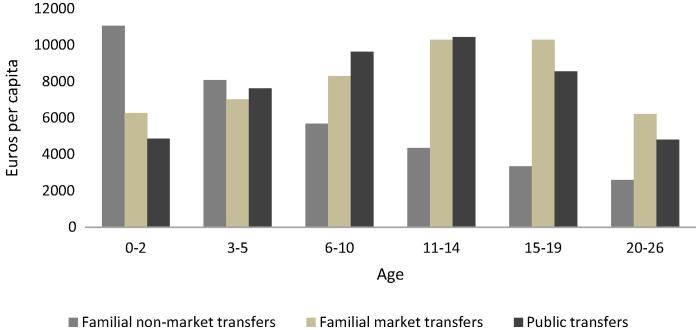
Support to young deficit ages by institutional and economic sector, Italy 2008.

*Source:* Own elaborations on Eurostat (2008a,b,c,d), Istat (2008a, 2009) and various other sources.

As already discussed, most of the life cycle deficit at young ages is financed by the family. Young persons continue to be net beneficiaries of private transfers even when they are already net payers in the public sector. Fig. 6 shows the support of young deficit ages by the institutional and economic sector, with the purpose to obtain an insight into the subsidiary redistributive function of Italian families. Young deficit ages (from 0 to 26 years) have been disaggregated according to the different stages of the Italian education system.^11^ Families’ transfers represent more than 50% of the total transfers received by children in all age groups considered. Time transfers are a significant component of familial support for all ages. Children aged from 0 to 2 years receive the highest amounts of familial transfers (about 78% of total transfers), of which the majority are in the form of time transfers. Public transfers for the 3–5 years age group are slightly increasing, mainly due to the greater availability of institutional care, mirrored in a decrease of familial transfers, in particular of time transfers. During school ages, public and private transfers are almost balanced due to public expenditure for education. However, with the end of compulsory schooling, net public transfers decrease meaning that again family resources become more important in determining and safeguarding the children’s possibilities.

Finally, Table 2 analyzes the effect of changes in population age structure on the deficit of the economic life cycle. Eurostat population projections (Eurostat, 2014b) have been used to derive values of life cycle deficit and surplus for the years 2030 and 2050, behind the (simplifying) assumption of constant labour income and consumption age profiles. During the whole period considered, as an effect of demographic change, the total deficit of the young would decrease by 5%, surplus would decrease by 17% and the deficit of the old would increase by 76%. The market and non-market economic sector highlight the existence, on the one hand, of a similar behaviour for young dependency and working-ages surplus, on the other, of significant differences at older ages. Old-age dependency would increase by more than 72% in the market economy but by less than 15% in the non-market economy, due to the active role of elderly persons in household production until very old ages. At the aggregate level the ageing of the population would cause a reduction of total production by 10%, in detail market production would be reduced by 20% while non-market production would even slight increase.

## Discussion and conclusions

This article represents a first attempt to develop a comprehensive account of monetary and time transfers between genders and generations for Italy. For this purpose an extensive database has been developed encompassing estimates for average production, consumption, transfer payments and benefits by single-year age group and gender for both the public and the private sector. Estimates concerning the private sector, i.e. families, include both market and non-market household production. The inclusion of unpaid work is of fundamental relevance within the Italian *familialistic* (Saraceno, 1997; Esping Andersen, 1999; Leitner, 2003) welfare system.

The cross-sectional picture of the Italian system of age- and gender-related economic transfers for 2008 revealed three main interconnected themes: (i) the existence of an important public transfer imbalance toward old ages; (ii) a prolonged youth dependency borne by families, both through monetary and time transfers; (iii) the permanence of a strong gender bias in the division of market and domestic work. Results seem to suggest the existence of both implicit gender and generational contracts: women take most of the responsibility for domestic work and family care while men focus on paid work, allowing for a market surplus during their working as well as retirement ages which is mostly used to support younger generations and women. Results for the non-market economy highlight the existence of a gender gap already at young ages and of a considerable amount of work performed by women during virtually all the lifetime. Furthermore, the existence of both a market and a non-market surplus during central ages may suggest the existence of a double shift (Hochschild, 1989) for women.

Results for the support system at young ages confirm that in Italy the responsibility for child care during early childhood is traditionally attributed to the family. The *familiarisation* of intergenerational obligations toward young ages poses several questions in terms of sustainability and equity of the Italian welfare system. On the one hand, care responsibilities are one of the principal reasons for the persistence of a strong gender specialization in production. Feminist reformers have strongly supported the provision of good quality institutional care not only as a fundamental tool in order to manage parenthood and market work but also as an essential prerequisite for the achievement of gender equality (Bergmann, 1986). On the other hand, the role of families in providing social welfare for adult children may facilitate the transmission of inherited privileges and thus the consequent reproduction of social inequalities (Livi Bacci, 2008).

Summarizing, the results from this article suggest the necessity for Italy to redesign social policies, rethinking intergenerational and gender balances on the basis of the current social needs and risks. In particular, among policy priorities, public intervention toward work-family reconciliation and *de-familiarisation* of responsibilities for children appears to be essential.

## Figures and Tables

**Table A1:** CHILD CARE: Parameter estimates.

Age	Parameter estimate	Standard error	*t* for *H*_0_: parameter = 0	*Pr* > |*t*|
0	13713	157	87.54	<0.0001
1	9083	135	67.50	<0.0001
2	7296	136	53.63	<0.0001
4	6032	137	43.88	<0.0001
4	5357	137	39.14	<0.0001
5	4376	142	30.88	<0.0001
6	3697	142	26.04	<0.0001
7	3606	138	26.14	<0.0001
8	3156	140	22.60	<0.0001
9	2931	131	22.30	<0.0001
10	2521	144	17.51	<0.0001
11	1653	132	12.51	<0.0001
12	1837	139	13.19	<0.0001
13	1219	141	8.67	<0.0001
14	1081	127	8.49	<0.0001
15	1103	130	8.47	<0.0001
16	512	128	4.00	<0.0001
17	669	130	5.15	<0.0001

**Table A2:** ADULT CARE: Parameter estimates.

Age	Parameter estimate	Standard error	*t* for *H*_0_: parameter = 0	*Pr* > |*t*|
18	534	191	2.80	0.0052
19	394	182	2.16	0.0308
20	182	179	1.01	0.3111
21	43	187	0.23	0.8189
22	706	191	3.69	0.0002
23	332	186	1.79	0.0736
24	131	182	0.72	0.4710
25	131	180	0.73	0.4661
26	15	180	0.09	0.9317
27	322	183	1.76	0.0781
28	268	176	1.52	0.1286
29	49	173	0.28	0.7773
30	373	171	2.18	0.0294
31	425	166	2.56	0.0106
32	9	152	0.06	0.9546
33	228	153	1.50	0.1347
34	151	146	1.04	0.2982
35	335	149	2.24	0.0250
36	246	145	1.69	0.0904
37	202	149	1.36	0.1738
38	342	145	2.36	0.0183
39	295	143	2.06	0.0390
40	319	141	2.27	0.0234
41	392	138	2.85	0.0044
42	33	139	0.24	0.8122
43	321	137	2.34	0.0194
44	383	137	2.79	0.0052
45	437	143	3.06	0.0022
46	101	144	0.70	0.4834
47	174	148	1.18	0.2390
48	242	156	1.55	0.1206
49	554	147	3.77	0.0002
50	531	153	3.48	0.0005
51	550	157	3.49	0.0005
52	419	153	2.74	0.0061
53	470	161	2.92	0.0035
54	541	153	3.53	0.0004
55	417	161	2.58	0.0098
56	360	162	2.23	0.0258
57	269	151	1.79	0.0736
58	269	156	1.72	0.0849
59	544	149	3.65	0.0003
60	449	149	3.01	0.0026
61	732	154	4.77	<0.0001
62	741	158	4.68	<0.0001
63	421	187	2.25	0.0242
64	258	177	1.46	0.1442
65	1386	166	8.35	0.0000
66	516	173	2.99	0.0028
67	536	155	3.46	0.0005
68	307	163	1.88	0.0598
69	215	161	1.34	0.1806
70	692	165	4.20	<0.0001
71	710	178	4.00	<0.0001
72	649	175	3.70	0.0002
73	870	174	5.00	<0.0001
74	1124	186	6.03	<0.0001
75	521	178	2.92	<0.0001
76	769	186	4.14	<0.0001
77	771	185	4.17	<0.0001
78	771	195	3.95	<0.0001
79	1421	203	7.00	<0.0001
80	1049	203	5.18	<0.0001
81	1163	214	5.43	<0.0001
82	1194	219	5.45	<0.0001
83	1234	251	4.92	<0.0001
84	1097	258	4.26	<0.0001
85	1363	269	5.07	<0.0001
86	1131	267	4.24	<0.0001
87	1575	311	5.06	<0.0001
88	2876	338	8.51	<0.0001
89	1020	465	2.19	0.0283
90	2587	209	12.35	<0.0001

**Table A3:** HOUSEWORK: Parameter estimates.

Age	Parameter estimate	Standard error	*t* for *H*_0_: parameter = 0	*Pr* > |*t*|
0	274	624	0.44	0.6613
1	144	540	0.27	0.7895
2	584	546	1.07	0.2845
3	5067	549	9.22	<0.0001
4	3311	544	6.63	<0.0001
5	3726	562	7.42	<0.0001
6	4165	561	8.43	<0.0001
7	4609	547	8.60	<0.0001
8	4755	553	8.60	<0.0001
9	4651	522	8.91	<0.0001
10	5589	571	9.79	<0.0001
11	3738	525	7.12	<0.0001
12	4567	553	8.26	<0.0001
13	6364	558	11.41	<0.0001
14	4916	507	9.69	<0.0001
15	5905	521	11.33	<0.0001
16	5890	514	11.45	<0.0001
17	4929	519	9.51	<0.0001
18	5315	538	9.89	<0.0001
19	6138	514	11.94	<0.0001
20	6010	506	11.88	<0.0001
21	5233	528	9.91	<0.0001
22	5585	539	10.36	<0.0001
23	7416	523	14.18	<0.0001
24	7807	513	15.22	<0.0001
25	7029	506	13.88	<0.0001
26	7558	508	14.88	<0.0001
27	7830	516	15.19	<0.0001
28	8695	497	17.49	<0.0001
29	6997	488	14.34	<0.0001
30	7443	483	15.40	<0.0001
31	9062	473	19.16	<0.0001
32	7643	433	17.66	<0.0001
33	8247	436	18.90	<0.0001
34	8137	418	19.45	<0.0001
35	8486	433	19.62	<0.0001
36	8781	420	20.93	<0.0001
37	8915	430	20.75	<0.0001
38	8137	419	19.41	<0.0001
39	8700	415	20.96	<0.0001
40	8799	408	21.55	<0.0001
41	9004	401	22.46	<0.0001
42	8958	403	22.25	<0.0001
43	8540	399	21.40	<0.0001
44	9422	399	23.64	<0.0001
45	8769	414	21.18	<0.0001
46	10335	412	25.06	<0.0001
47	9749	425	22.95	<0.0001
48	9716	447	21.73	<0.0001
49	9269	418	22.18	<0.0001
50	10539	433	24.35	<0.0001
51	10251	444	23.07	<0.0001
52	10188	432	23.59	<0.0001
53	11063	454	24.36	<0.0001
54	10124	432	23.42	<0.0001
55	10618	455	23.36	<0.0001
56	9713	455	21.36	<0.0001
57	10650	424	25.12	<0.0001
58	11601	439	26.40	<0.0001
59	12615	419	30.09	<0.0001
60	12099	419	28.87	<0.0001
61	12520	432	28.95	<0.0001
62	11618	445	26.09	<0.0001
63	12219	526	23.23	<0.0001
64	12269	498	24.65	<0.0001
65	12131	467	25.96	<0.0001
66	13089	486	26.94	<0.0001
67	12386	436	28.41	<0.0001
68	12608	459	27.48	<0.0001
69	12483	452	27.59	<0.0001
70	12749	464	27.47	<0.0001
71	13023	500	26.05	<0.0001
72	12102	494	24.51	<0.0001
73	13207	489	27.00	<0.0001
74	13252	524	25.28	<0.0001
75	11841	502	23.58	<0.0001
76	12165	523	23.28	<0.0001
77	12027	520	23.13	<0.0001
78	11304	549	20.60	<0.0001
79	11561	571	20.24	<0.0001
80	11765	570	20.64	<0.0001
81	11037	602	18.32	<0.0001
82	10389	616	16.85	<0.0001
83	9794	707	13.86	<0.0001
84	10342	725	14.27	<0.0001
85	10678	756	14.13	<0.0001
86	9160	751	12.20	<0.0001
87	8860	876	10.12	<0.0001
88	8335	951	8.76	<0.0001
89	8507	1308	6.51	<0.0001
90	5924	589	10.06	<0.0001

**Table 1:** Aggregate values of LCD and LCS by gender and economic sector.

Economy	Deficit of the young			Surplus ages			Deficit of the elderly		
	Male	Female	Total	Male	Female	Total	Male	Female	Total
*Millions of Euro*									
Market	96991	104051	201042	−200086	−4411	−204497	102453	160523	262976
Non market	44199	28729	72928	35756	−88725	−52969	12971	−32929	−19958
Total	141190	132780	273970	−164330	−93136	−257466	115424	127594	243018
*Relative values*									
Non market/total	31	22	27	−22	95	21	11	−26	−8

*Source:* Own elaborations on Banca d’Italia (2008), Eurostat (2008a,b,c,d), Istat (2008a,b, 2009) and various other sources.

**Table 2:** Projected values of LCD and LCS for year 2030 and 2050 by economic sector.

Economic sector	Deficit and surplus ages	Absolute values		Values relative to year 2008	
		2030	2050	2030	2050
Market	Deficit of the young	196605	190385	98	95
	Surplus ages	−189492	−171299	93	84
	Deficit of the elderly	367029	451595	140	172
Non market	Deficit of the young	66226	65496	95	94
	Surplus ages	−46146	−42300	88	81
	Deficit of the elderly	−22623	−20109	129	114
Total economy	Deficit of the young	262831	255881	97	95
	Surplus ages	−235638	−213600	92	83
	Deficit of the elderly	344406	431485	140	176

*Source:* Own elaborations on Banca d’Italia (2008), Eurostat (2008a,b,c,d), Istat (2008a,b, 2009) and various other sources.

## Appendix Linear models for consumption of time use productive activities

A1. Linear model for consumption of child care activities

$${\text{CHILD}}_{j}=\overset{17}{\underset{x=0}{\sum }}\alpha (x)\cdot N_{j}(x)$$

where

CHILD*_j_* = time for childcare produced/consumed by household *j*;
*N_j_*(*x*) = number of members aged *x* in household *j*;
*α*(*x*) = parameters to be estimated.

A2. Linear model for consumption of adult care activities

$${\text{ADULT}}_{j}=\overset{90+}{\underset{x=18}{\sum }}\beta (x)\cdot N_{j}(x)$$

where

ADULT*_j_* = time for adult care produced/consumed by household *j*;
*N*_*j*_(*x*) = number of members aged *x* in household *j*;
*β*(*x*) = parameters to be estimated.

A3. Linear model for consumption of housework activities

$${\text{HOUSEWORK}}_{j}=\overset{90+}{\underset{x=18}{\sum }}\gamma (x)\cdot N_{j}(x)$$

where

HOUSEWORK*_j_* = time for housework produced/consumed by household *j*;
*N*_*j*_(*x*) = number of members aged *x* in household *j*;
*γ*(*x*) = parameters to be estimated.

## Footnotes

1 Per-capita age profiles are mainly based on sample estimates. These estimates can be noisy due to the sample errors, particularly at ages with relatively few observations. However, in some cases, significant age variations are not an effect of sample errors but reflect the actual trend by age of the considered phenomenon. This is the case of the very high levels of health consumption for newborns compared with that of subsequent ages. Another example is public consumption for education, where expenditures are significantly age-targeted and can be subject to important changes from one year to another, reflecting changes in spending related to the transition to different levels of education. In all these cases the age profiles have not been smoothed.

2 EU-SILC statistics for 2009 refer to the year 2008.

3 The analysis builds on data from the individuals and the daily diary files.

4 Diaries for pre-school children are filled by their parents. Household members are supposed to fill in the diary within the same day.

5 Age limits for child care and adult care were established on the basis of TUS definitions of children and adults, respectively.

6 Parameter estimates are obtained through the ordinary least squares method (OLS).

7 Sample weights are used to reproduce the demographic structure of the Italian population.

8 Information on labour income is reported in EU-SILC only from age 15, reflecting the Italian regulation on the minimum age to access the labour market.

9 Eurostat estimates the unadjusted Gender Pay Gap (GPG) as the difference between average gross hourly earnings of male and female paid employees as a percentage of the average gross hourly earnings of male paid employees. The population consists of all paid employees in enterprises with 10+ employees.

10 Deficit and surplus ages in Table 1 refer to those for total population.

11 Nursery from 0 to 2 years, day care from 3 to 5 years, primary school from 6 to 10 years, lower secondary from 11 to 13 years, upper secondary from 14 to 18 years, tertiary from 19 years.
